# The path of ambivalence: tracing the pull of opposing evaluations using mouse trajectories

**DOI:** 10.3389/fpsyg.2015.00996

**Published:** 2015-07-17

**Authors:** Iris K. Schneider, Frenk van Harreveld, Mark Rotteveel, Sascha Topolinski, Joop van der Pligt, Norbert Schwarz, Sander L. Koole

**Affiliations:** ^1^Mind and Society Center, University of Southern CaliforniaLos Angeles, CA, USA; ^2^Department of Psychology, University of Southern CaliforniaLos Angeles, CA, USA; ^3^Emotion Regulation Lab, Department of Clinical Psychology, VU University AmsterdamAmsterdam, Netherlands; ^4^Department of Psychology, University of AmsterdamAmsterdam, Netherlands; ^5^Department of Psychology, University of CologneCologne, Germany

**Keywords:** ambivalence, evaluation, embodiment, attitudes, mixed feelings

## Abstract

Ambivalence refers to a psychological conflict between opposing evaluations, often experienced as being torn between alternatives. This dynamic aspect of ambivalence is hard to capture with outcome-focused measures, such as response times or self-report. To gain more insight into ambivalence as it unfolds, the current work uses an embodied measure of pull, drawing on research in dynamic systems. In three studies, using different materials, we tracked people’s mouse movements as they chose between negative and positive evaluations of attitude objects. When participants evaluated ambivalent attitude objects, their mouse trajectories showed more pull of the non-chosen evaluative option than when they evaluated univalent attitude objects, revealing that participants were literally torn between the two opposing evaluations. We address the relationship of this dynamic measure to response time and self-reports of ambivalence and discuss implications and avenues for future research.

## Introduction

Imagine walking across campus after a long day of work. While you are heading to the train station, you notice a fast-food truck. While your preference for the ease of a quick and tasty snack might pull you toward the truck, your aversion to unhealthy foods may pull you away from it. And even though you keep heading for the train, your final path may show a curve, reflecting the pull of the fast-food truck.

The psychological state of being torn between opposing evaluations, or ambivalence, is an inescapable part of human life. People can be ambivalent about a wide range of topics including fast food, abortion, out-group members, organ donation, euthanasia, and alcohol (for overviews, see Sparks et al., 2001; van Harreveld et al., 2009b, 2014). Ambivalence exerts a pervasive influence on people’s emotions (e.g., van Harreveld et al., 2009a) and shapes how people process information and solve problems (e.g., Maio et al., 1996; Jonas et al., 1997; Nordgren et al., 2006; Rees et al., 2013; van Harreveld et al., 2014).

Conceptually, ambivalence involves a psychological conflict between opposing implicit or explicit evaluations about an attitude object (e.g., Meehl, 1964 [in Emmons, 1996]; Kaplan, 1972; Eagly and Chaiken, 1993; Thompson et al., 1995; Wegener et al., 1995; Priester and Petty, 1996; van Harreveld et al., 2009b; Petty et al., 2012). Ambivalence is thus not the same as feeling neutral or indifferent toward an attitude object, but is characterized by simultaneously having strong positive and negative associations (de Liver et al., 2007). The idea of conflict between evaluations implies that ambivalence is inherently dynamic and the resolution of the conflict is a process that evolves over time.

How might behavioral scientists be able to assess this psychological tug-of-war between opposing evaluations? Most indices of ambivalence are either assessed after an evaluation (i.e., response time based measures) or rely on people’s subjective assessment of their psychological state (i.e., self-report based measures), both of which may have their limitations. Whereas the former presents us with the problem of a black box (i.e., we cannot know what goes on during the formation of the response), the latter relies on people’s (often biased) insight into their own thoughts and feeling. Below, we discuss both approaches in turn.

Self-report based measures of ambivalence ask how conflicted individuals feel (e.g., Priester and Petty, 1996) or calculate ambivalence from separate positive and negative evaluations (e.g., Kaplan, 1972; Thompson et al., 1995; Larsen et al., 2009) or the number of positive and negative thoughts people are able to report about an ambivalent topic (e.g., van Harreveld et al., 2014). As such, they are informative about people’s subjective evaluations and the conflict between them. However, people may lack insight into their own thoughts and feelings. Consequently, self-report based measures may not tap into implicit forms of ambivalence (e.g., Petty et al., 2012). Moreover, assessing ambivalence through self-report based indices may cause the construct to become salient and thereby change the experience or evaluative process (see Schneider et al., unpublished manuscript). Finally, explicit measures may be affected by cultural and social factors that render people reluctant to explicitly report their ambivalence (e.g., Cavazza and Butera, 2008), especially on controversial matters such as abortion or euthanasia.

Researchers have also inferred ambivalence from response times (e.g., Fazio et al., 1986; Bargh et al., 1992), bypassing the problems associated with self-report. For instance, slower evaluation times may be interpreted as indicative of the cognitive competition between opposing evaluations because opposing evaluations take longer to integrate (Bargh et al., 1992; van Harreveld et al., 2004; Petty et al., 2006). The benefits of this approach are that it takes into account the influence of both implicit and explicit evaluations and does not rely on peoples’ subjective assessments of, and evaluations about, their ambivalence. However, response times only provide information after an evaluation is complete. As such, they do not inform researchers about the preceding stages of the evaluation process that lead to faster or slower responding (e.g., Schwarz and Bohner, 2001). It is therefore difficult to make strong claims about what underlies prolonged response times. For instance, slower responses may be the result of decreased accessibility (e.g., Fazio et al., 1986; Higgins, 1996), consideration of alternatives, behavioral inhibition due to conflict (Gray and McNaughton, 2000; see also Corr, 2013), or even decreased positive affect (Kuhl and Kazen, 1999). As a consequence, very little is known about how ambivalence unfolds during evaluation.

Thus, although ambivalence can be understood as a tug-of-war between evaluations, to our knowledge, no research has systematically addressed the dynamic evaluative processes that underlie ambivalence. The present research begins to fill this gap. Drawing on an approach developed in dynamic systems research, we propose that motor outputs during the evaluation process may provide a window into the unfolding of ambivalent evaluations.

### Evaluation and Motor Output

Research has shown that motor output can readily reveal people’s evaluative stance toward attitude objects. For instance, subtle changes in specific muscles in the face (e.g., Cacioppo et al., 1986) or in posture (Hillman et al., 2004; Eerland et al., 2012; Schneider et al., 2013) can reveal positive and negative evaluations toward an attitude object (see also Niedenthal et al., 2005). However, not only the end state, but also the *unfolding* of evaluations as they form is observable in overt motor behavior. During the formation of an attitude, associated evaluations rise and fall in activation until one holds sway long enough to become the final judgment (Wojnowicz et al., 2009). Because the sensorimotor circuits responsible for *executing* actions are also involved in *deciding which* action to take, information from high level processing overflows to pre motor cortices (Cisek and Kalaska, 2010). Thus, whenever an evaluation is dominant, even for a short period of time, the accompanying motor response is also activated, and visible in overt behavior (e.g., Freeman et al., 2011).

As a consequence, ongoing mental processes can be observed in even simple behaviors, such as mouse movements on a computer screen. In the mouse tracking paradigm people engage in a computer task in which they perform certain actions, for instance categorization of human faces as male or female, by moving their mouse toward specific response locations on the screen. While they do so, the path of their mouse is traced and recorded. The characteristics of the trajectories, such as its curvature for instance, can be used to give insight into evaluative conflict *while* people are performing their task (Freeman and Ambady, 2010, for an overview see Freeman et al., 2011), resembling our opening example of the walking trajectory resulting from the pull of the food truck and the avoidance of unhealthy snacks. Such an approach has not been used in ambivalence research, but it has been successfully applied in other domains.

For instance, in stereotyping research, when people were asked to evaluate whether masculine or female faces fitted a specific trait by dragging the mouse over the screen to a certain response, arm movements showed pull to both responses when the face was *both* masculine and feminine (Freeman and Ambady, 2009). In other words, people’s movements while choosing between the stereotypical female and the stereotypical male traits shown on screen revealed the conflict between femininity and masculinity in response to the stimulus. Likewise, mouse trajectories have been used to reveal implicit racial bias: When people indicated they ‘liked’ (vs. ‘disliked’) black people, peoples arm movements moved both to the dislike response and the like response when ‘liking’ black people, but less so when they indicated they ‘liked’ white people (Wojnowicz et al., 2009).

### The Present Studies

Capitalizing on the embodied expression of ongoing evaluations, we conducted three studies in which we employed a mouse tracker paradigm, allowing us to assess motor output during an evaluative task. In accordance with previous operationalizations of cognitive competition (Wojnowicz et al., 2009; Freeman and Ambady, 2010) we used the extent to which the curvature of participants’ mouse trajectory deviated toward the unselected response (Maximum Deviation, MD, cf. Wojnowicz et al., 2009) as a measure of pull of opposing evaluations. To illustrate, a univalent topic with *only* negative or only positive evaluations associated with it, is expected to yield a relatively straight line from the start button to the response button. An ambivalent topic with *both* negative and positive evaluations is expected to show a line that is partially pulled to both response options, creating a more curved line. This measure of pull is indicative for the degree of conflict and cognitive competition between evaluations (e.g., Freeman and Ambady, 2010). We expected that pull of the opposing evaluation (as measured by MD) would be greater for ambivalent compared to univalent attitude objects. Such a finding would not only add to the literature on ambivalence, showing that ambivalent evaluation can be captured in an embodied way, but also add to the mouse tracking literature by extending the paradigm into the domain of attitudinal ambivalence.

It is worth noting that one earlier study has related mouse locations on a computer screen to assess mixed feelings toward a target person (Vallacher et al., 1994). Although at first sight, this may seem similar to the mouse tracking paradigm used here, the procedure relied on participants’ insight in their feelings from moment to moment, reflecting the end products of repeated evaluations. More specifically, participants were instructed to use the location of the mouse pointer relative to a target on screen to indicate changes in their explicit evaluation with regard to a target person. Over a period of 2 minutes, participants were to move closer to the target when they felt more positive and move further away when they felt more negative. This pioneering work showed that when people have mixed feelings toward target person, they more often change locations, and this does not diminish over time. This indeed captures one aspect of the dynamic nature of ambivalence, namely, attitude inconsistency. In other words, these findings show that people’s ambivalent attitudes give rise to different evaluations at different time points. Instead, in the present research, we looked at motor output that does not rely on introspection and assesses conflict as it dynamically unfolds *during* evaluation.

Aside from our primary measure of pull, we also assessed response times. As noted above, previous work found that people take longer to evaluate ambivalent attitude objects, compared to non-ambivalent attitude objects (e.g., Bargh et al., 1992; van Harreveld et al., 2004; de Liver et al., 2007). Measuring response times allowed us to explore possible correlations to online processes of ambivalence, self-report measures, and response times. For exploratory purposes we also looked at the moment in time in which pull was largest.

In all studies, we report all manipulations, all participants and exclusions, and all dependent measures. To reduce the influence of outlier responses, latencies under 300 ms as well as above 3,000 ms were excluded from analyses in all studies, however, including these observations did not alter our results. Cohen’s *d*_z_ effect sizes (standardized mean difference between two groups of dependent observations, Cohen, 1988) and 95% confidence intervals around the effect size are calculated using a procedure developed by Wuensch (2012). Additionally, we also report $\eta_{p}^{2}$. For all experiments, the number of participants was determined by the amount of scheduled lab time that was available to the experimenter, but was always at least 30 participants. This number gave us enough power to detect small to medium effects in complete within subjects design experiments.

## Study 1

### Method

#### Participants and Design

Forty-nine students (34 females, 3 unreported, *M*_age_ = 22, SD_age_ = 3) participated for course credit or monetary reward. The experiment followed a one-factor (valence: ambivalent vs. univalent) within-subjects design with pull (operationalized as the degree to which the curvature of the trajectory deviated toward the unselected response, or MD) as main dependent variable. All participants signed an informed consent form. In the same session, we also collected data involving evaluations of then current Dutch politicians and affectively laden pictures, which are not related to the current project and not reported further.

#### Attitude Objects

We used 12 attitude objects that have successfully been used in prior research to induce ambivalence (de Liver et al., 2007; Schneider et al., 2013; van Harreveld et al., 2014). The ambivalent topics were: abortion, organ donation, euthanasia, and alcohol. The univalent topics were: happy, holiday, in love (in Dutch this is *one* word), sunshine, abuse, depressed, disgust, and unhappy. Initially we also included four attitude objects with which we had no previous experience. These attitude objects were: me, future, sports, and blood donation. However, after inspection of explicit ratings, we decided not to include these attitude objects in the current analyses, as they were not rated as ambivalent by our participants.

#### Evaluation Task

Upon entering the lab, participants were seated in individual cubicles. All instructions were computer-administered. In the first part of the experiment, participants were asked to evaluate different words as positive or negative. Participants were instructed to respond as quickly and accurately as possible. At the start of each trial, a start button appeared at the bottom center of the screen and the response options (positive and negative) appeared in the top left and top right corner of the screen. When participants clicked the start button at the center bottom of the screen, the cursor was relocated to the center starting position (to ensure all trajectories start from the same location) and the attitude object appeared. Participants then moved the mouse to one of the two response buttons located in the top right and top left of the screen. After this, the attitude object disappeared and a new trial started.

At the start of the experiment, participants completed two neutral practice trials to familiarize themselves with the task. Each participant evaluated each attitude object twice in two separate blocks of trials, including 12 attitude objects each, resulting in 24 trials. In each block, all attitude objects were presented once, in a random order. The location of the positive and the negative response option was reversed between blocks. Unfortunately, due to a technical error, only one counterbalancing condition functioned, and block order was not counterbalanced. For all participants, in the first block, the positive response option appeared on the right and the negative on the left. In the second block, this location was reversed and the negative response option appeared on the left and the positive response option on the right.

During each trial, streaming *x* and *y* coordinates of the computer mouse were recorded at a sampling rate of approximately 70 Hz. Participants’ response times (the time between stimulus onset and final click) were also unobtrusively recorded. To record, process, and analyze mouse trajectories, we used the validated MouseTracker software package, which includes modules for recording and analyzing mouse trajectories reliably (Freeman and Ambady, 2010). All trajectories were rescaled into a standard coordinate space (top left: *x*, *y* = [1, 1.5]; bottom right: *x*, *y* = [1, 0]). Because trials differed in their length, raw trajectories were normalized into 101 time steps using linear interpolation so that all trials had the same amount of time steps and could be averaged. To obtain an index of the degree to which there was pull toward the eventually non-chosen evaluation, we calculated MD as follows. First, an idealized response trajectory (i.e., a straight line between each trajectory’s start point and endpoint) was computed. The MD was then calculated as the largest perpendicular deviation between the realized trajectory and its idealized trajectory. MDs were then averaged over stimuli, trials, and conditions. Higher MDs indicated more deviation from the idealized trajectory toward the unselected alternative and more pull. Apart from MD, it is also possible to measure the area under the curve (AUC), which refers to the geometric area between the actual trajectory and the ideal trajectory (i.e., straight line) and is also an index of pull. In general, for the same data, MD and AUC do not give substantially different results (Freeman and Ambady, 2010). In line with this, assessing AUC instead of MD did not alter the results of our studies, and AUC and MD showed a strong positive correlation (*r* = 0.97, *p* < 0.001 for Study 1, *r* = 0.96, *p* < 0.001, in Study 2, and *r* = 0.90, *p* < 0.001, in Study 3), thus in the remainder of the manuscript we present results for MD only.

#### Ambivalence

Ambivalence was measured with three established measures of ambivalence. First, *objective* ambivalence was measured using two items assessing participants’ negative and positive evaluations of each particular word and image independently (Kaplan, 1972). We asked participants to ignore their negative (vs. positive) evaluations and rate how positive (vs. negative) they thought the word/image was. The items read: “Think about [stimulus word]. When you think about the positive (negative) aspects, while ignoring the negative (positive) aspects, how positive is your evaluation of this?” Participants gave their ratings on a scale ranging from “*not at all positive (negative)*” to “*very positive (negative)*.” We calculated objective ambivalence using the following formula: [(*P* + *N*)/*2*] – |*P* – *N*| (Thompson et al., 1995). Participants’ scores on the objective ambivalent scale could range from – 1.5 to 4, where higher scores reflect more ambivalence.

Second, participants’ *subjective* ambivalence was measured with one item: “To what degree do you experience conflicting thoughts and/or feelings regarding this?” The nine-point answer scales ranged from “*no conflicting thoughts and feelings at all*” to “*maximum conflicting thoughts and feelings*” (cf. Priester and Petty, 1996).

Third, we measured participants’ *affective* ambivalence toward the attitude objects using the evaluative space grid (ESG: Larsen et al., 2009). The evaluative space grid allows participants to indicate their positive and negative evaluations on a 5 × 5 grid, using the *X*-axis to indicate positivity and the *Y*-axis to indicate negativity. The question on the *X*-axis was: “How positive do you feel about this”? The question on the *Y*-axis was: “How negative do you feel about this?” The cells on the axes were labeled as “*not at all positive* (vs. *negative*)” to “*very positive* (vs. *negative*).” Overall scores were calculated with the formula: [(*P* + *N*)/*2*] *–* |*P* – *N*|. Participants’ scores on the ESG could range from – 1 to 5, with higher scores reflecting more ambivalence in affective responses toward the objects.

### Results and Discussion

Due to a technical error, the data of one participant were lost. One participant did not provide any self-report ratings. To reduce the influence of outlier responses, latencies under 300 ms as well as above 3,000 ms (1.9% of trials; cf. Bargh et al., 1992; de Liver et al., 2007) and errors (i.e., positive responses to negative stimuli and negative responses to positive stimuli, 0.9% of trials) were removed from the dataset.

#### Ambivalence

Paired sample *t*-tests showed that for objective ambivalence, ambivalent stimuli had higher scores (*M* = 1.76 SD = 0.81) than univalent stimuli (*M* = 0.11, SD = 0.54), *t*(47) = 13.90, *p* < 0.001, $\eta_{p}^{2}$ = 0.80, Cohen’s *d*_z_ = 2.01, 95% CI [1.51–2.50]. The same was true for subjective ambivalence (*M* = 5.28, SD = 1.43 vs. *M* = 2.32, SD = 1.40), *t*(47) = 12.65, *p* < 0.001, $\eta_{p}^{2}$ = 0.77, Cohen’s *d*_z_ = 1.83, 95% CI [1.36–2.29] and affective ambivalence (*M* = 1.63, SD = 1.05 vs. *M* = –0.32, SD = 0.65), *t*(47) = 13.63, *p* < 0.001, $\eta_{p}^{2}$ = 0.80, Cohen’s *d*_z_ = 1.97, 95% CI [1.48–2.45].

#### Pull

**FIGURE 1:**
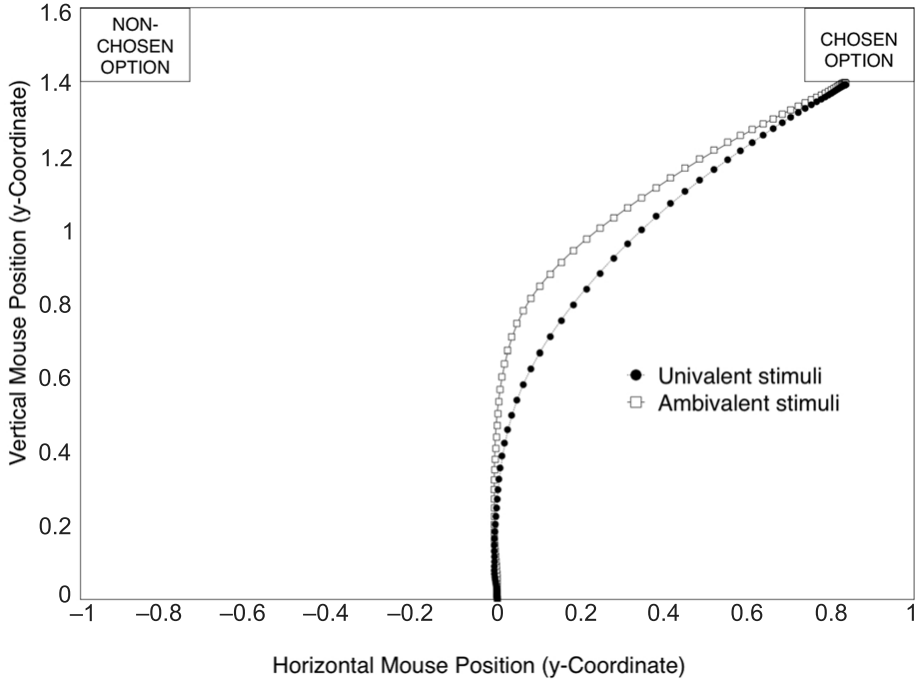
**Mean mouse trajectories in the evaluation task, averaged over ambivalent and univalent stimuli.** In this figure, all trajectories are mapped rightward to allow comparison.

As predicted, participants’ mouse trajectories showed a greater pull toward the opposite evaluation when they evaluated ambivalent (*M* = 0.55, SD = 0.31) rather than univalent stimuli (*M* = 0.33, SD = 0.23), *t*(48) = 6.19, *p* < 0.001, $\eta_{p}^{2}$ = 0.44, Cohen’s *d*_z_ = 0.88, 95% CI [0.55, 1.21], resulting in a larger curve (see **Figure 1**)

Moreover, the moment at which the pull of the opposing evaluation was largest, occurred later for ambivalent stimuli (*M* = 832.75, SD = 182.78) than for univalent stimuli (*M* = 702.74, SD = 128.79), *t*(48) = 5.92, *p* < 0.001, $\eta_{p}^{2}$ = 0.42, Cohen’s *d*_z_ = 0.85, 95% CI [0.52, 1.17].

#### Response Times

Responses were faster in the second than in the first block; however, the pattern of results was the same and we collapsed the data over blocks. Replicating numerous previous findings (e.g., Fazio et al., 1986; Bargh et al., 1992; van Harreveld et al., 2004), participants were slower when they evaluated ambivalent (*M* = 1475.31, SD = 220.36) rather than univalent stimuli (*M* = 1270.63, SD = 176.01), *t*(48) = 6.59, *p* < 0.001, $\eta_{p}^{2}$ = 0.48, Cohen’s *d*_z_ = 0.94, 95% CI [0.60, 1.28]. More important, response times did not correlate with pull, *r*(49) = -0.076, *p* = 0.604, and when differences in pull between ambivalent and univalent stimuli were entered as a covariate, the effect of ambivalence on response times remained significant, *F*(1,47) = 7.04, *p* = 0.011 This suggests that the longer reaction times observed for ambivalent stimuli are not solely attributable to the pull between evaluations and may also be influenced by other factors.

#### Additional Analyses

The measure of pull did not correlate with self-report measures of ambivalence. Because we orthogonally manipulated ambivalence, perhaps reducing variance, we separately examined correlations between our measures for ambivalent and univalent stimuli. These analyses did not reveal significant relations between the response times and pull on the one side, and self-report measures of ambivalence on the other side. This is surprising because earlier work (e.g., Bargh et al., 1992) has found correlations between objective ambivalence and response times, although these studies did not manipulate, but rather measured ambivalence.

Finally, exploratory analyses showed that for ambivalent stimuli, trajectories showed more directional reversals over both the horizontal axis (i.e., x-flips; *M* = 6.82, SD = 1.55 vs. *M* = 6.21, SD = 1.33, respectively, *t*(47) = 2.76, *p* = 0.008, $\eta_{p}^{2}$ = 0.17, Cohen’s *d*_z_ = 0.40, 95% CI [0.10, 0.69]) and the vertical axis of the screen (i.e., y-flips; *M* = 5.89, SD = 1.55 vs. *M* = 5.04, SD = 1.23, respectively, *t*(47) = 4.01, *p* < 0.001, $\eta_{p}^{2}$ = 0.28, Cohen’s *d*_z_ = 0.58, 95% CI [0.27, 0.88]) indicating that they were somewhat more complex (Freeman and Ambady, 2010). Although both x-flips (*r* = 0.49, *p* < 0.001) and y-flips (*r* = 0.68, *p* < 0.001) correlated with our measure of pull, entering them as covariates did not alter our patterns of results and the effect of valence on pull remained intact.

### Conclusion

As hypothesized, during evaluation of ambivalent attitude objects, pull toward the non-chosen evaluation was greater than during the evaluation of univalent attitude objects, indicating that ambivalence literally pulls people in two directions. Shedding more light on the unfolding of ambivalence during evaluation, we also found that maximum conflict occurred later in time for ambivalent evaluations, compared to univalent evaluations. Additionally, we replicated the effect of ambivalence on response times (Fazio et al., 1986; Bargh et al., 1992; van Harreveld et al., 2004). However, response time did not correlate with amount of pull nor with self-report measures of ambivalence. Self-report also did not correlate with pull. This could mean that slower responses to ambivalent stimuli do not only reflect the conflict associated with ambivalence. We will return to this matter in the Section “General Discussion.” Finally, exploratory analyses showed ambivalent stimuli elicited more reversals in trajectories than univalent stimuli, a possible indication that evaluating these stimuli is more complex.

## Study 2

Study 2 replicates and extends the first study by examining the process of evaluating health-related ambivalent attitude objects. Unhealthy eating and drinking behavior are known to be strong elicitors of ambivalence (Sparks et al., 2001; Conner and Sparks, 2002); therefore we used palatable but unhealthy foods and alcohol as ambivalent attitude objects and healthy foods and orange juice as univalent attitude objects in Study 2. These ambivalent and univalent stimuli are highly and similarly familiar, which allowed us to rule out an alternative explanation of Study 1, namely, that the ambivalent topics used there (e.g., euthanasia) were simply harder to bring to mind than the univalent topics (e.g., sunshine) and hence caused slower responses (c.f. Schwarz and Bohner, 2001).

### Method

#### Participants and Design

Thirty-eight students (28 females, *M*_age_ = 22, SD_age_ = 6) participated for course credit or monetary reward. The experiment was run at the end of an experimental hour and was preceded by unrelated tasks. The experiment followed a one-factor (valence; ambivalent vs. univalent) within-subjects design, with pull (MD) as main dependent variable. All participants signed an informed consent form.

#### Attitude Objects

In this study, we used images depicting different kinds of foods and beverages (for clarity we will refer to ‘foods’ for this category throughout the manuscript). The ambivalent attitude objects depicted: beer, a hamburger, chocolate, and fries. The univalent attitude objects depicted vegetables, an apple, bread, and orange juice, resulting in a total of eight trials. The attitude objects were presented in random order. The images were acquired from the Internet and, keeping original constraints, scaled to 300 pixels wide and presented against a white background.

#### Measures and Procedure

The experiment was administered in the same manner as Study 1, using the same measures, with the exception that the location of the response buttons on the screen was counterbalanced between participants. All attitude objects were presented only once and no habituation could ensue. Finally, the neutral practice trials consisted of an image of baskets and an image of a gray desk cabinet.

### Results and Discussion

Responses under 300 ms and above 3,000 ms were removed from the data (2.6%) as well as errors (i.e., negative responses to the generally positive univalent stimuli, 1.6%; cf. de Liver et al., 2007). Normalization and calculation of MD was done in the same manner as in Study 1.

#### Ambivalence

Ambivalence scores were higher for ambivalent attitude objects than for univalent attitude objects on objective ambivalence (*M* = 1.77, SD = 1.01 vs. *M* = 0.51, SD = 0.79, respectively), *t*(37) = 7.19, *p* < 0.001, $\eta_{p}^{2}$ = 0.58, Cohen’s *d*_z_ = 1.17, 95% CI [0.75, 1.58], subjective ambivalence (*M* = 4.64, SD = 1.68 vs. *M* = 2.34, SD = 1.33, respectively), *t*(37) = 7.26, *p* < 0.001, η^2^ = 0.59, Cohen’s *d*_z_ = 1.18, 95% CI [0.76, 1.59], and affective ambivalence (*M* = 1.44, SD = 1.01 vs. *M* = –0.12, SD = 0.71, respectively), *t*(37) = 8.36, *p* < 0.001, $\eta_{p}^{2}$ = 0.65, Cohen’s *d*_z_ = 1.36, 95% CI [0.91, 1.79].

#### Pull

Confirming our hypotheses, we found that pull was greater for ambivalent attitude objects (*M* = 0.44, SD = 0.32) than for univalent attitude objects (*M* = 0.25, SD = 0.25), *t*(37) = 2.98, *p* = 0.005, $\eta_{p}^{2}$ = 0.19, Cohen’s *d*_z_ = 0.48, 95% CI [0.14, 0.82]. Moreover, the moment at which the pull of the opposing evaluation was largest, occurred later for ambivalent stimuli (*M* = 868.27, SD = 263.62) than for univalent stimuli (*M* = 674.30, SD = 187.84), *t*(37) = 5.65, *p* < 0.001, $\eta_{p}^{2}$ = 0.46, Cohen’s *d*_z_ = 0.92, 95% CI [0.53–1.29]. These observations replicate Study 1.

#### Response Times

The data revealed slower responses for ambivalent stimuli (*M* = 1517.70, SD = 325.43) compared to univalent stimuli (*M* = 1251.47, SD = 245.05), *t*(37) = 6.05, *p* < 0.001, $\eta_{p}^{2}$ = 0.50, Cohen’s *d*_z_ = 0.98, 95% CI [0.59–1.37]. However, the response times did not correlate with pull, *r*(38) = 0.082, *p* = 0.624, and after entering the difference in pull as a covariate, the effect of valence on response times remained in tact, *F*(1,36) = 22.23, *p* < 0.001. These observations further replicate Study 1.

#### Additional Analyses

Examining the correlations between the mouse tracker variables and the self-report variables, we found that pull was positively correlated with subjective ambivalence *r*(38) = 0.40, *p* = 0.012 and objective ambivalence, *r*(38) = 0.38, *p* = 0.021. Similar correlations were found between response time and subjective ambivalence *r*(38) = 0.38, *p* = 0.020, and objective ambivalence, *r*(38) = 0.40, *p* = 0.012. Thus, self-reported based ambivalence correlated positive with both pull and response time. However, pull and response times were not correlated with each other *r*(38) = 0.082, *p* = 0.624, indicating that they may be influenced by different aspects. Exploratory analyses showed that there were no differences in reversals over either axes between ambivalent and univalent stimuli, although both x-flips (*r* = 0.45, *p* = 0.005) and y-flips (*r* = 0.50, *p* = 0.001) correlated with our measure of pull.

### Conclusion

The results of Study 2 replicated the core findings of Study 1. Ambivalent objects were associated with stronger pull indicating more ambivalence. Additionally, ambivalent and univalent stimuli did not only differ in amount of pull and magnitude of curves, but also in the temporal unfolding. Finally, pull and response time were not correlated.

However, the pattern of correlations between self-reported and pull and response times differed from Study 1, such that subjective and objective ambivalence did have a positive relationship with both the magnitude of pull as well as the time people took to respond. These differences across studies may be due to differences between the stimuli used (abortion and euthanasia in Study 1; foods in Study 2). To test this possibility we directly compared the two types of stimuli in one design in Study 3.

## Study 3

In Study 3, we used the attitude objects from Study 2 (foods) and Study 1 (abstract topics) in a single design. To make sure that possible differences were not due to mode of presentation (i.e., Study 1 stimuli presented as words vs. Study 2 stimuli presented as images) we now presented the attitude objects from Study 2 also as words. Thus, instead of showing a picture of a hamburger, we now presented the word “hamburger.” Second, because one of the attitude objects in the category foods was “beer,” we decided to drop “alcohol” from the Study 1 attitude objects, to prevent stimulus overlap.

### Method

#### Participants and Design

Forty-five students (29 females, 1 unreported, *M*_age_ = 21, SD_age_ = 3) participated for course credit or monetary reward. The experiment followed a two-factor (valence; ambivalent vs. univalent, topic; foods vs. abstract) within subjects design with pull (MD) as main dependent variable. All participants signed an informed consent form.

#### Attitude Objects

In this experiment, we used attitude objects from both previous studies, resulting in 17 trials (7 ambivalent, 10 univalent, see below). From Study 1, we took the ambivalent abstract topics: abortion, euthanasia, and organ donation (we had only three stimuli here, because we dropped alcohol from this category, as it would already appear in the food stimuli category as “beer”). The univalent abstract topics were: depressed, abuse, unhappy, happy, sun, and holiday. We used the same food stimuli as in Study 2. The ambivalent food stimuli were: beer, hamburger, chocolate, and fries with sauce^1^. The univalent food stimuli were: vegetables, apple, bread, and orange juice (as in Study 2).

#### Measures and Procedure

The experiment was run in the same manner as Studies 1 and 2, using the same measurements.

### Results and Discussion

One participant did not provide data on the self-report measures. Responses given under 300 ms as well as above 3,000 ms were removed from the data (3.5%) as well as errors (i.e., positive responses to negative stimuli and negative responses to positive stimuli, 0.4%; cf. de Liver et al., 2007). Normalization and calculation of MD was done in the same manner as in Studies 1 and 2.

#### Ambivalence

Ambivalent attitude objects were more ambivalent compared to univalent attitude objects for objective ambivalence, *F*(1,43) = 110.36, *p* < 0.001, $\eta_{p}^{2}$ = 0.72, Cohen’s *d*_z_ = 1.55, 95% CI [1.11, 1.98], subjective ambivalence, *F*(1,43) = 112.54, *p* < 0.001, $\eta_{p}^{2}$ = 0.72, Cohen’s *d*_z_ = 1.60, 95% CI [1.15, 2.04], and affective ambivalence, *F*(1,43) = 168.68, *p* < 0.001, $\eta_{p}^{2}$ = 0.80, Cohen’s *d*_z_ = 1.96, 95% CI [1.45, 2.56], see **Table 1** for means. There was also an interaction between topic and valence, such that for the affective ambivalence measure, the effect was stronger for abstract topics, compared to foods, *F*(1,43) = 25.39, *p* < 0.001, $\eta_{p}^{2}$ = 0.37. There was no main effect of topic.

**Table 1:** Study 3 mean ambivalent ratings for objective, subjective and affective ambivalence for ambivalent vs. univalent stimuli, for abstract topics, foods, and overall.

	Objective Ambivalence		Subjective Ambivalence		Affective Ambivalence	
	Ambivalent	Univalent	Ambivalent	Univalent	Ambivalent	Univalent
Overall	1.51 (0.87)	0.18 (0.62)	3.74 (1.34)	2.02 (0.86)	1.23 (0.98)	–0.16 (0.64)
Abstract topics	1.59 (0.92)	0.13 (0.63)	3.77 (1.95)	2.20 (1.35)	1.43 (1.12)	–0.46 (0.71)
Foods	1.43 (1.13)	0.23 (0.77)	3.72 (1.85)	1.84 (0.86)	1.03 (1.21)	0.13 (0.74)

#### Pull

Repeated measures analyses with valence (ambivalent vs. univalent) and topic (abstract vs. foods) as within-subjects factor and MD as dependent variable showed a main effect of topic and valence (see **Table 2** for relevant means). Results also showed an interaction between topic and valence. As expected, pull was stronger for ambivalent attitude objects, compared to univalent attitude objects, *F*(1,44) = 67.98, *p* < 0.001, $\eta_{p}^{2}$ = 0.61, Cohen’s *d*_z_ = 1.24, 95% CI [0.85–1.63]. This means that participants showed stronger pull toward the non-choice option for the ambivalent attitude objects (*M* = 0.48, SD = 0.25) compared to the univalent attitude objects (*M* = 0.19, SD = 0.17). The moment of greatest pull also occurred later in time for ambivalent attitude objects (*M* = 951.60, SD = 236.80) compared to the univalent attitude objects (*M* = 704.97, SD = 140.28), *F*(1,44) = 121.76, *p* < 0.001, $\eta_{p}^{2}$ = 0.74, Cohen’s *d*_z_ = 1.65, 95% CI [1.19–2.09] again indicating a temporal difference in the unfolding of ambivalence. These observations replicate Studies 1 and 2.

**Table 2:** Study 3 means (SD) for maximum deviation (MD), response time (RT), and maximum deviation time (MDT) for ambivalent vs. univalent stimuli, for abstract topics and foods separately.

	Abstract Topics		Foods	
	Ambivalent	Univalent	Ambivalent	Univalent
MD	0.70 (0.38)	0.23 (0.21)	0.26 (0.24)	0.14 (0.23)
RT	1737.01 (354.71)	1237.29 (206.13)	1388.71 (306.11)	1244.66 (263.83)
MDT	1063.11 (286.33)	682.58 (128.29)	840.08 (244.26)	727.35 (185.61)

The main effect of topic revealed that pull was greater for abstract topics (*M* = 0.47, SD = 0.25) compared to foods (*M* = 0.20, SD = 0.19), *F*(1,44) = 40.50, *p* < 0.001, $\eta_{p}^{2}$ = 0.48, Cohen’s *d*_z_ = 0.95, 95% CI [0.59–1.30] and that the moment of greatest pull occurred later in time for ambivalent abstract topics (*M* = 872.85, SD = 183.47) compared to the foods (*M* = 783.72, SD = 203.64), *F*(1,44) = 16.81, *p* < 0.001, $\eta_{p}^{2}$ = 0.28, Cohen’s *d*_z_ = 0.61, 95% CI [0.30–0.93].

Finally, there was an interaction between valence and topic such that the main effect of valence was also somewhat stronger for abstract topics, compared to foods, *F*(1,44) = 31.23, *p* < 0.001, $\eta_{p}^{2}$ = 0.42. With regard to moment of occurrence of greatest pull, the interaction indicated that in general people were slower for abstract topics than food when it came to ambivalent stimuli, but this pattern was not statistically significant by conventional standards (*p* = 0.055) for the univalent stimuli (a similar pattern was found for response times overall, see below), *F*(1,44) = 40.75, *p* < 0.001, $\eta_{p}^{2}$ = 0.48.

#### Response Times

A main effect of valence showed slower responses for ambivalent stimuli (*M* = 1562.85, SD = 291.46) compared to univalent stimuli (*M* = 1240.98, SD = 224.32), *F*(1,44) = 170.40, *p* < 0.001, $\eta_{p}^{2}$ = 0.80, Cohen’s *d*_z_ = 1.95, 95% CI [1.44, 2.44]. This replicates Studies 1 and 2 as well as earlier research. There was also a main effect of topic, showing that people were slower in responding to the abstract topics (*M* = 1487.15, SD = 255.33) than to food stimuli (*M* = 1316.68, SD = 270.88), *F*(1,44) = 38.50, *p* < 0.001, $\eta_{p}^{2}$ = 0.48, Cohen’s *d*_z_ = 0.93, 95% CI [0.57–1.27]. Finally, there was also an interaction between topic and valence, *F*(1,44) = 52.49, *p* < 0.001, $\eta_{p}^{2}$ = 0.54, qualifying the main effect of topic such that for ambivalent stimuli, people were slower for abstract topics, but for univalent stimuli there was no such difference.

#### Additional Analyses

Overall, response times correlated marginally negatively with pull, *r*(45) = –0.28, *p* = 0.066. As in Studies 1 and 2, entering differences in MD as a covariate still left an effect of valence on RT, *F*(1,43) = 50.49, *p* < 0.001. Furthermore, the data showed a positive correlation between subjective ambivalence and amount of pull, *r*(44) = 0.34, *p* = 0.024, but no other correlations were statistically significant. When conducting the same analyses on abstract topics and foods separately, there were no significant correlations between the measures. Exploratory analyses showed that for trajectories for ambivalent stimuli showed more directional reversals over both the horizontal axis (i.e., x-flips; *M* = 7.03, SD = 1.81 vs. *M* = 6.44, SD = 1.74, respectively, *F*(1,44) = 5.41, *p* = 0.025, $\eta_{p}^{2}$ = 0.11, Cohen’s *d*_z_ = 0.47, 95% CI [0.16, 0.78]) and the vertical axis of the screen (i.e., y-flips; *M* = 5.75, SD = 1.74 vs. *M* = 5.23, SD = 1.38, respectively, *F*(1,44) = 10.13, *p* < 0.001, $\eta_{p}^{2}$ = 0.19, Cohen’s *d*_z_ = 0.49, 95% CI [0.17, 0.79]) indicating that they were somewhat more complex (Freeman and Ambady, 2010). Although x-flips correlated with the measure of pull (*r* = 0.32, *p* = 0.03), entering these, or y-flips, as covariates did not alter our patterns of results and the effect of valence on pull remained intact.

#### Food vs. Abstract Topics

Although, overall, we obtained the same pattern of results for both foods and abstract topics, effect sizes between studies differed. To investigate whether these signify a structural difference between these topic domains we compared size of the effect of our manipulation for foods vs. abstract topics for all our measures. First, we calculated effect sizes for foods and abstract topics separately in Study 3. We then calculated a point estimate for each index and topic domain separately and tested whether the point estimates differed significantly (see **Table 3**). These analyses showed that there was a statistically significant difference in effect sizes for pull (MD) over studies, such that effects were stronger for abstract topics compared to food. There was a marginally significant difference on the measure of objective ambivalence, showing stronger effects for abstract topics.

**Table 3:** Point estimates [95% CI] for effect sizes for objective, subjective, and affective ambivalence, response times (RT), curvature (MD), and moment of occurrence of maximum conflict (MD time).

	Abstract Topics	Foods	*t*	*df*	*p*
Objective ambivalence	1.66 [1.11, 2.22]	1.08 [0.81, 1.34]	3.47	1	0.06
Subjective ambivalence	1.35 [0.50, 2.19]	1.09 [0.82, 1.36]	0.33	1	0.57
Affective ambivalence	1.62 [1.02, 2.21]	1.37 [0.73, 2.01]	0.32	1	0.57
RT	1.32 [0.47, 2.18]	0.85 [0.60, 1.10]	1.06	1	0.30
MD	1.05 [0.66, 1.44]	0.45 [0.23, 0.68]	6.64	1	0.01
MD time	1.15 [0.50, 1.80]	0.81 [0.56, 1.05]	0.94	1	0.33

*Effect sizes pooled over topic domain (abstract topics vs. foods).*

#### Pictures vs. Words

In addition, the data of Study 2 and the food conditions of Study 3 can be brought to bear on whether pictures of foods or words describing foods are more likely to elicit ambivalence. Although both studies presented the same attitude objects, the visual presentation of foods caused stronger effects on the subjective measure of ambivalence, (point estimate = 1.75, 95% CI [1.28, 2.22]) than the presentation of food words (point estimate = 1.08, CI [0.71, 1.46], *t*(1) = 4.77, *p* = 0.029. None of the other indices showed differences in effect size (all *p*’s > 0.47).

### Conclusion

As expected, we found that evaluating ambivalent attitude objects, compared to univalent attitude objects, caused more pull toward the non-chosen option, both for abstract topics and foods. Additionally, the moment of maximum pull occurred later for ambivalent attitude objects. These findings replicate the effect found in Studies 1 and 2. We also replicated the effect of valence on response times – ambivalent evaluations took longer. Pull was also positively correlated with subjective ambivalence. Comparing effect sizes across studies, we found that when it comes to different topic domains, abstract topics yield larger effect sizes on pull of opposing evaluations and objective ambivalence, but not on any of the other indices. Finally, pictures of foods elicit somewhat stronger effects on subjective ambivalence compared to food words, but not on any of the other indexes of ambivalence.

## General Discussion

Ambivalence is a psychological state in which people are being torn between “one side” and the “other side” when making evaluations. This dynamic aspect of ambivalence is hard to capture in response times or self-report based measures of ambivalence. To gain more insight into ambivalence as it unfolds, we used an embodied measure of pull, drawing on research on dynamic systems (Freeman and Ambady, 2009, 2010; Wojnowicz et al., 2009; Freeman et al., 2011). In three studies, we tracked participants’ mouse movements as they evaluated attitude objects that varied in ambivalence.

### Summary of Main Findings

Several findings are worth highlighting. First, our data show that amount of pull as assessed with the embodied measure, is sensitive to differences in ambivalence. When participants evaluated an ambivalent attitude object, their mouse trajectories showed more pull of the non-chosen option than when they evaluated a univalent attitude object, revealing how they were literally torn between two opposing evaluations. Second, the temporal trajectory of the motor movements suggests that the peak of experienced conflict, as indexed by the moment of maximum pull, occurs later for ambivalent than for non-ambivalent attitude objects. Thus, our work shows not only a difference in degree of pull between opposing evaluations, but also sheds light on its temporal unfolding. Finally, across all three studies, we replicated earlier findings that evaluating ambivalent attitude objects takes more time overall than evaluating non-ambivalent attitude objects (Bargh et al., 1992; van Harreveld et al., 2004). Notably, these differences in response time remain, even when controlling for the conflict between opposing evaluations as measured by pull, indicating that slower evaluations may not be exclusively due to evaluative conflict.

Within cognitive psychology, longer response times have been considered an indicator of cognitive competition and conflict (e.g., MacLeod, 1991). Ambivalence researchers have followed this convention, by assuming that longer response times to ambivalent stimuli arise from competing evaluative responses, which also need to be integrated for a final answer (Bargh et al., 1992; Bassili, 1996). Because we found variation in the correlations between pull and response time, our findings suggests that slower responses times may be, at least in part, driven by other factors including consideration of a broader set of attributes that may reduce the conflict and increased elaboration (cf. Schwarz and Bohner, 2001; Schwarz, 2007), behavioral inhibition due in response to conflict (Gray and McNaughton, 2000; Corr, 2013) or decreased positive affect (Kuhl and Kazen, 1999).

### Future Research

In the present studies ambivalence was not experimentally manipulated and, instead, we used pre-selected materials. One might argue that our stimuli also differed on other dimensions than just ambivalence, for instance familiarity. However, our materials were all based on previous research into ambivalence and have been tried and tested in an array of studies (Sparks et al., 2001; van Harreveld et al., 2009a, 2014; Schneider et al., 2013). Furthermore, the measure of pull we used is a very close operationalization of the theoretical conceptualization of ambivalence in the literature. More importantly, participants indicated being ambivalent about our stimuli, making ambivalence at least a salient difference, albeit possibly not the only one. Thus, we believe that our results are very much tied to differences in ambivalence. Nevertheless, future research could experimentally manipulate ambivalence, by for instance introducing new (neutral) attitude objects that vary only on this dimension. Such research may provide additional empirical insight into convergent and divergent validity of mouse tracking measure for the purpose of assessing ambivalence.

It is worth noting that we found some variation in the relationship between pull and self-report based measures of ambivalence. Ambivalence is theoretically conceptualized as a state of evaluative conflict, caused by simultaneous activation of opposing evaluations (e.g., Meehl, 1964 [in Emmons, 1996]; Kaplan, 1972; Eagly and Chaiken, 1993; Thompson et al., 1995; Wegener et al., 1995; Priester and Petty, 1996; van Harreveld et al., 2009b; Petty et al., 2012). As noted above, our measure of pull presumably taps into exactly this conflict (cf. Freeman and Ambady, 2010, for an in depth discussion of mouse tracking). However, the variation in correlations with self-reports suggests that people are not always aware of the dynamic tug-of-war between opposing evaluations. For instance, in Study 2 and 3, measures of pull correlated positively with subjective ambivalence, such that the stronger the experience of ambivalence, the stronger the pull. However this correlation was not statistically significant in Study 1. Furthermore, objective ambivalence related significantly to pull only in Study 2. Based on this, we believe that the mouse trajectory measure taps in to the subjective experience of ambivalence mostly, albeit not always. This raises the question which factors influence the strength of the relationship between self-report based and implicit measures of ambivalence, and under what conditions measures of pull correlate with self-report based measures, and under which they do not.

Previous work has addressed a similar question with regard to relatively low correlations between different self-report based measures of ambivalence. Measures of subjective and objective ambivalence generally show only moderate correlations (e.g., Thompson et al., 1995; Priester and Petty, 1996) and the magnitude of this correlation varies over studies. Addressing this, researchers found that correlations between the two are high when ambivalence is made salient (a situational factor), or when participants have a strong preference for consistency (an individual difference variable), (Newby-Clark et al., 2002). Similar avenues of research may focus on when self-report based measures of ambivalence correlate strongly with implicit indices, and when they do not (see also Koole et al., 2001, on the relation between implicit and explicit measures in the domain of self-esteem), and to what degree they relate to established findings in the literature on the consequences of ambivalence.

A notable finding from our exploratory analyses showed that, at least for Studies 1 and 3, trajectories were more complex as a function of ambivalence. This suggests that online experiences of ambivalence are an important factor in the driving and altering of response planning and execution. Future work might extend these preliminary findings and further understanding of the embodied consequences of ambivalence. It is also worth exploring whether different types of ambivalent attitudes unfold differently over time. Conceptually, different types of ambivalence can be distinguished. Intracomponent ambivalence refers to ambivalence as the result of conflicting cognitions (cognitive/cognitive conflict) or conflicting affective responses (affective/affective conflict), (van Harreveld et al., 2009b). Intercomponent ambivalence, or affective–cognitive ambivalence, refers to ambivalence as the result of conflict between cognitions and affective responses (Lavine et al., 1998; Maio et al., 2000). Another distinction can be made between ambivalence based on mostly “cold” arguments or more “hot” affective responses, or even mixed emotions (i.e., emotional ambivalence; e.g., Larsen et al., 2001; Fong, 2006; Rees et al., 2013). Paralleling this, ambivalent trajectories might unfold differently as a result of individual differences (e.g., self-control and conflicted feelings toward certain foods; Gillebaart et al., submited).

Our work shows that ambivalence is readily captured in mouse movements. Together with previous work on whole body movement and ambivalence (Schneider et al., 2013), this suggests that ambivalence has an overt visible quality. Work on grasping behaviors has shown that observers are able differentiate actors intentions based on the kinematics of the actors’ movement (Becchio et al., 2008, 2012). Relating this to the current findings, observers may be able to infer actors’ ambivalence, purely from watching their body movements.

Finally, given that ambivalence has important consequences for information processing (e.g., Maio et al., 1996; Jonas et al., 1997; Fong, 2006; Nordgren et al., 2006; Rees et al., 2013; van Harreveld et al., 2014) and behavior (e.g., Lipkus et al., 2001; Priester, 2002; Berndsen et al., 2004; Costarelli and Colloca, 2004), the question becomes whether and when pull or self-report based measures of ambivalence better predict these outcomes. Research has shown for instance, that implicit ambivalence has consequences for behavior, even though people do not report on it (Petty et al., 2012). In view of these findings, future research may examine if amount of pull is a better predictor of downstream consequences of ambivalence than self-reported ambivalence.

## Conclusion

In the present work, we took a first step in assessing ambivalence through an embodied measure of pull between opposing evaluations. We found that the measure of pull is sensitive to manipulations of ambivalence, showing that people are torn between evaluations when evaluating an ambivalent attitude object. Pull was mostly positively correlated with self-reports of subjective ambivalence. However, it was not consistently related to objective ambivalence and response time, suggesting that there is more to ambivalence than these measures capture. Future research should further address the degree to which the measure of pull differentially relates to self-report measures and predicts different outcomes. The embodied approach used here adds to the methodological repertoire that researchers can draw on to further unravel the dynamic and multifaceted nature of ambivalence.

## Conflict of Interest Statement

The authors declare that the research was conducted in the absence of any commercial or financial relationships that could be construed as a potential conflict of interest.

## Footnotes

1 In the Netherlands, it is customary to eat fries with some sort of sauce. Mayonnaise is usually the sauce of choice (in fact, to the degree that “fries *with*,” would automatically denote mayonnaise), but other common favorites include, but are not limited to, “special” (mixture of mayonnaise, ketchup, and raw onions), “war” (mixture of mayonnaise, peanut sauce, and raw onions), and “joppie” (secret recipe, but presumably a mixture of red curry, yellow curry, mayonnaise, sugar, and raw onions). Because some people do not like mayonnaise, but instead prefer for instance, peanut sauce or ketchup, we chose to use the addition “with sauce” to mean fries with some form of relatively greasy sauce, regardless of the precise contents, but applicable to the preference of our participants.
